# Attosecond streaking using a rescattered electron in an intense laser field

**DOI:** 10.1038/s41598-020-79034-2

**Published:** 2020-12-16

**Authors:** Yang Hwan Kim, Igor A. Ivanov, Sung In Hwang, Kyungseung Kim, Chang Hee Nam, Kyung Taec Kim

**Affiliations:** 1 Center for Relativistic Laser Science, Institute for Basic Science, Gwangju, 61005 Korea; 2 Department of Physics and Photon Science, Gwangju Institute of Science and Technology, Gwangju, 61005 Korea

**Keywords:** Ultrafast photonics, Nonlinear optics

## Abstract

When an atom or molecule is exposed to a strong laser field, an electron can tunnel out from the parent ion and moves along a specific trajectory. This ultrafast electron motion is sensitive to a variation of the laser field. Thus, it can be used as a fast temporal gate for the temporal characterization of the laser field. Here, we demonstrate a new type of attosecond streaking wherein a rescattered electron trajectory is manipulated by an ultrashort laser pulse. The vector potential of the laser pulse is directly recorded in the photoelectron spectra of the rescattered electron. In contrast to high harmonic generation methods, our approach has no directional ambiguity in space, leading to complete in situ temporal characterization. In addition, it provides timing information on ionization and re-scattering events. Therefore, our approach can be a useful tool for the investigation of strong-field processes triggered by rescattering, such as non-sequential double ionization and laser-induced electron diffraction.

## Introduction

The temporal characterization of a laser field is of great interest in many laser applications. There are many temporal characterization techniques that can be applied for femtosecond laser pulses such as FROG and SPIDER^1–3^. They utilize a response from a nonlinear crystal for the temporal characterization of a laser pulse. The complete characterization of the laser field including the carrier-envelope phase (CEP) has also been demonstrated using the attosecond streaking technique^4,5^. Recently, it was also shown that the laser pulse can be characterized using ionization in the air^6^. These pulse characterization techniques are convenient for use in many applications. However, as they require particular nonlinear interactions, they are in general implemented independently.

For the temporal characterization of ultrashort laser pulses used for laser–matter interactions, it is ideal to measure a laser pulse at the place where it is being used. A few approaches support such in situ measurements utilizing the ultrafast electron dynamics. They utilize the process of high-harmonic generation (HHG) as a fast temporal gate^7–11^, which is particularly useful for the characterization of the laser field used in high harmonic spectroscopy. However, there is ambiguity of the direction of the reconstructed laser field since the relative phase between two laser pulses is measured. The direction of the laser field in space cannot be determined by these measurements.

In this work, we demonstrate a new attosecond streaking technique in which a laser field is completely determined by manipulating the trajectory of a backward rescattered electron in an intense laser field. Since the electron dynamics in the process of above-threshold ionization (ATI) is utilized, it can be applied for a broad intensity range of ${10}^{13}\sim {10}^{15}$ W/cm^2^. The direction of the laser field can be completely determined without ambiguity thanks to the stereo-ATI time-of-flight spectrometer used for the photoelectron measurement. In addition, our new streaking technique provides timing information on ionization and backward rescattering events. Therefore, it will become a useful tool for the investigation of physical processes related to rescattering dynamics such as such as HHG^12^, ATI^13,14^, non-sequential double ionization^15–17^, and laser-induced electron diffraction^18–22^.

## Results

**Figure 1:**
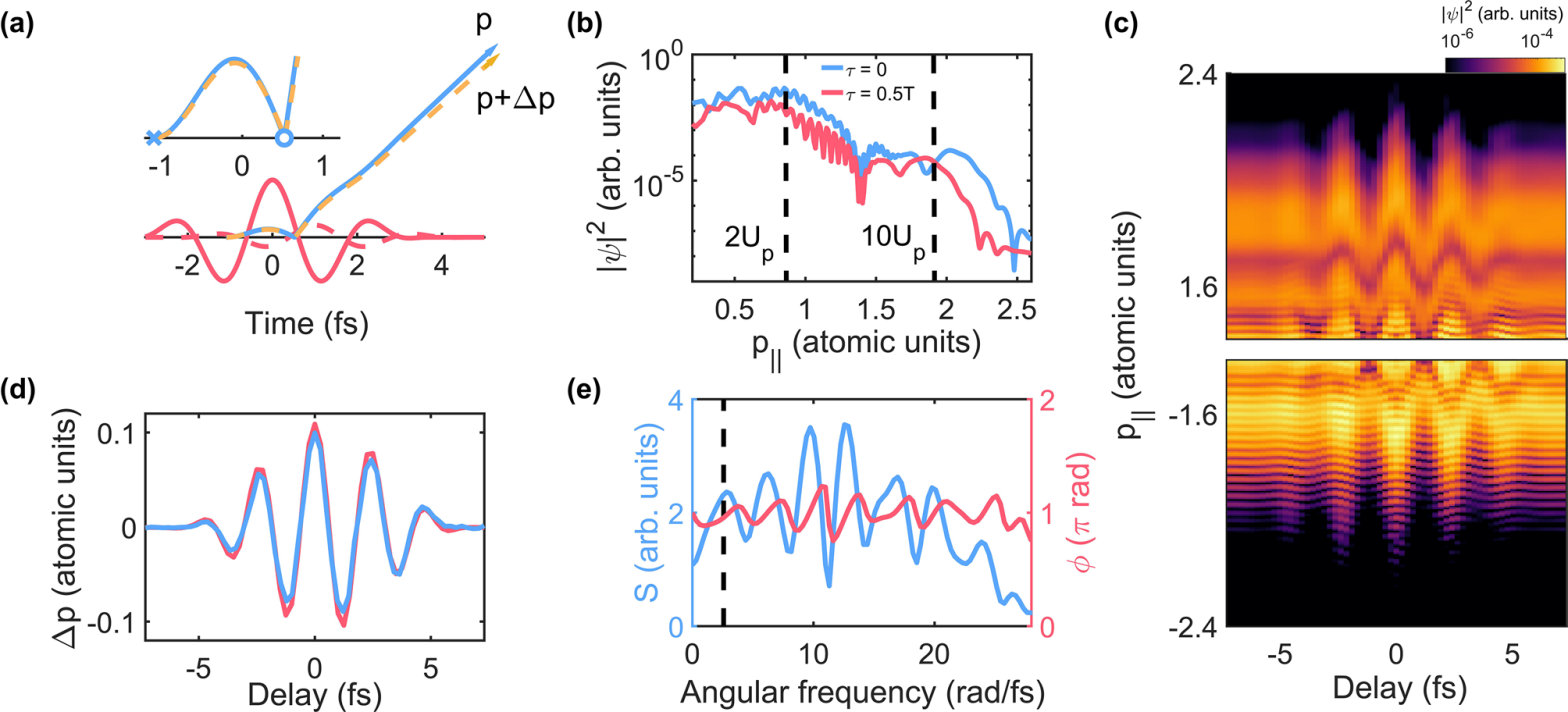
Manipulation of a backward rescattered electron trajectory in above-threshold ionization. (**a**) Classical electron trajectories that yield maximum energy along one direction in an intense laser field with (orange dashed arrow) or without (blue solid arrow) a perturbing laser field. The main laser field and the perturbing laser field are indicated by the red solid line and red dashed line, respectively. The magnified plot from the ionization time (blue cross) to the backward rescattering time (blue circle) is shown in the inset. (**b**) ATI spectra along the polarization direction of the laser field obtained at zero (blue solid line) and half-cycle (0.5 T, red solid line) delays between the main laser field and the perturbing laser field. (**c**) ATI spectra along the polarization of the laser field obtained by solving the time-dependent Schrodinger equation in 3D with a model potential of Xe using the main pulse with a peak intensity of $1 \times 10^{14}$ W/cm^2^ and pulse duration of 4.4 fs together with the perturbing pulse, which has the same temporal waveform as the main field with a peak intensity of $5.0 \times 10^{11}$ W/cm^2^. The CEPs of both pulses were $0.5\pi$. (**d**) The cutoff momentum shift $\Delta p\left( \tau \right)$ obtained by fitting the Airy function to the spectra shown in (**c**) (blue solid line) and $\Delta p\left( \tau \right)$ obtained by solving the semi-classical model ignoring Coulomb field (red solid line). (**e**) Spectral response of the streaking method estimated by solving TDSE calculations. The vertical dashed line indicates the center angular frequency ${\omega }_{0}$ of a main pulse. A 730 nm, 5 fs laser pulse with the intensity of $2.0 \times 10^{14}$ W/cm^2^ was used for the main laser pulse. A single-cycle laser pulse with the center frequency of 8 ${\omega }_{0}$ was used as a perturbing field.

We used the semi-classical model based on strong-field approximation^12^ to describe the dynamics of an electron in an intense laser field. In the semi-classical model, an electron moves along different classical trajectories depending on its ionization time^23^. We focus on a backward rescattered electron that gains the maximum energy (called the cutoff electron hereafter). The cutoff electron tunnels out near the local extrema of the electric field and is driven back to the parent ion as the direction of the laser field is reversed, as shown in Fig. 1a. Then, the electron is rescattered backward near the next zero-crossing time of the laser field.

The trajectory of the cutoff electron can be manipulated by adding a weak perturbing laser field^24,25^. The intensity of the perturbing laser field is significantly weaker than that of the main pulse (see the Methods section). The addition of the perturbing laser field changes the final momentum of the cutoff electron by Δ*p* as shown in Fig. 1a. The cutoff momentum shift $\Delta p$ can be found as a function of the time delay $\tau$ between the main and perturbing laser pulses using the semi-classical model (see the Methods section for derivation),

1 $$\Delta p\left(\tau \right)=-2{A}_{p}\left({t}_{r}-\tau \right)+\frac{1}{{t}_{r}-{t}_{i}}{\int }_{{t}_{i}}^{{t}_{r}}{A}_{p}\left(t-\tau \right)\mathrm{d}t,$$

where ${A}_{p}$ is vector potential of the perturbing laser field, and ${t}_{i}$ and ${t}_{r}$ are ionization time and backward rescattering time in the absence of the perturbing field, respectively. The first term on the right-hand side of Eq. (1) is proportional to the vector potential of the perturbing field at the rescattering time and the second term is proportional to the time-averaged value of the vector potential of the perturbing field between the ionization time and backward rescattering time. Therefore, the vector potential of the perturbing laser field, ionization time, and backward rescattering time can be found in the momentum shift of the cutoff electron $\Delta p(\tau )$ (see the Methods section for details).

The cutoff momentum shift $\Delta p(\tau )$ of a backward rescattered electron can be found by analyzing the above-threshold ionization spectrum. While the cutoff energy of an electron directly liberated or rescattered forward reaches only up to $2{U}_{p}$, the cutoff energy of the backward rescattered electron reaches up to approximately $10{U}_{p}$ as shown in Fig. 1b. Here, ${U}_{p}$ is ponderomotive energy of an electron in the laser field. Therefore, the spectrum of the backward rescattered electron is clearly distinguished from that of the direct or forward rescattered electrons. However, the cutoff momentum shift $\Delta p(\tau )$ should be estimated carefully because the perturbing field alters the momentum and tunneling ionization rate at the same time. For example, photoelectron spectra obtained at two time delays by solving the time-dependent Schrödinger equation (TDSE) in 3D for a Xe potential exhibit differences in the amplitude and cutoff momentum as shown in Fig. 1b,c. Therefore, the cutoff momentum shift cannot be simply determined, and both the cutoff momentum shift and the amplitude modulation should be taken into account.

It is known that the momentum distribution of a backward rescattered electron along the polarization of the laser field in the cutoff energy region is well described by the Airy function^26^. Therefore, we fit the Airy function to the ATI spectra at each delay to find the cutoff momentum shift. To validate our approach, we compared the cutoff momentum shift $\Delta p(\tau )$ obtained by fitting the Airy function to the ATI spectra shown in the upper panel of Fig. 1c, with the cutoff momentum shift $\Delta p(\tau )$ obtained by solving the semi-classical model, as shown in Fig. 1d. The cutoff momentum shift induced by the perturbing field was accurately retrieved by fitting the Airy function to the ATI spectra.

The cutoff momentum shift in Eq. (1) can be averaged out if the frequency of the perturbing laser field is too high. This limits the frequency of the perturbing laser field that can be measured. This spectral response was estimated by solving TDSE using a single-cycle perturbing laser field whose beandwidth is broader than $10{\omega }_{0}$. The spectral amplitude and phase of the cutoff modulation show a broad distribution up to $10{\omega }_{0}$ as shown in Fig. 1e where ${\omega }_{0}$ is the center angular frequency of the main pulse. It means that our streaking method can be applied for a broad spectral range up to $10{\omega }_{0}$. The spectral phase modulates within $\pm 0.2\pi$ rad only, indicating that the cutoff modulation is similar to the temporal profile of the perturbing laser field. This spectral modulation was reproduced using Eq. (1). Therefore, the temporal profile of the perturbing laser field can be accurately reconstructed using Eq. (1).

**Figure 2:**
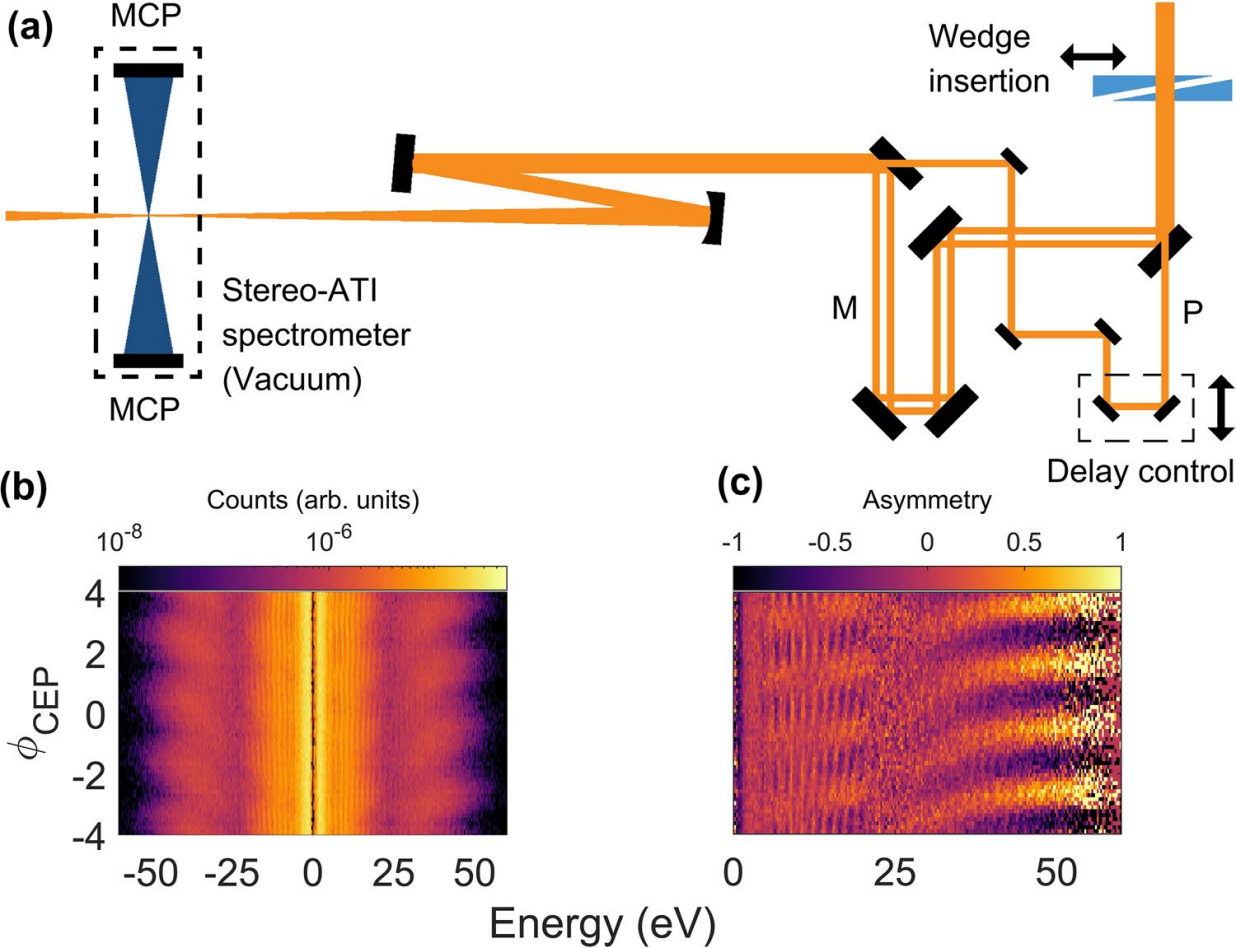
Schematic drawing of the experimental setup and CEP dependence of the momentum distribution. (**a**) Schematic drawing of the experimental setup. The input laser pulse is split into a main pulse (denoted by M) and a perturbing pulse (denoted by P) using a mirror with a hole at the center. The two laser pulses are combined after controlling the time delay (see the Methods section). (**b**) Stereo-ATI spectra recorded using the main laser pulse with a duration of 5 fs and a peak intensity of $1\times {10}^{14}$ W/cm^2^ in Xe for different CEPs. The negative sign in energy is for denoting direction (left) opposite to the positive sign (right). (**c**) Left–right asymmetry obtained from (**b**).

For the experimental demonstration, we measured ATI spectra using a homebuilt stereo-ATI time-of-flight spectrometer^27^ as shown in Fig. 2a (see the Methods section). The pulse was split into two pulses (denoted by M: main pulse, and by P: perturbing pulse, as shown in Fig. 2a) and combined with a time delay $\tau$ using a mirror with a round hole at the center in an interferometer without any dispersive optical elements. In this manner, the two pulses have the same waveform while the peak intensity of the perturbing pulse was only 0.5% of the main pulse at the focus. ATI spectra for both left and right directions along the polarization direction were measured using the stereo-ATI spectrometer. Slits were placed in the stereo-ATI spectrometer to obtain an energy spectrum with a small opening angle (2°) along the polarization of the laser field. The target cylinder (not shown in Fig. 2) of the spectrometer was filled with Xe gas with a pressure of $2.0\times {10}^{-4}$ torr. We scanned the insertion of the wedge pair before we scan the delay. Corresponding stereo-ATI spectra and asymmetry plot are shown in Fig. 2b,c. We chose specific CEPs of $-0.46\pi$ and $0.54\pi$ for delay scans so that the continuum at the cutoff is observed for both directions. The continuum spectrum indicates that the cutoff spectrum is contributed by electrons tunneled out from a single half optical cycle of the laser field. Therefore, Eq. (1) can be directly applied for the cutoff momentum shift.

**Figure 3:**
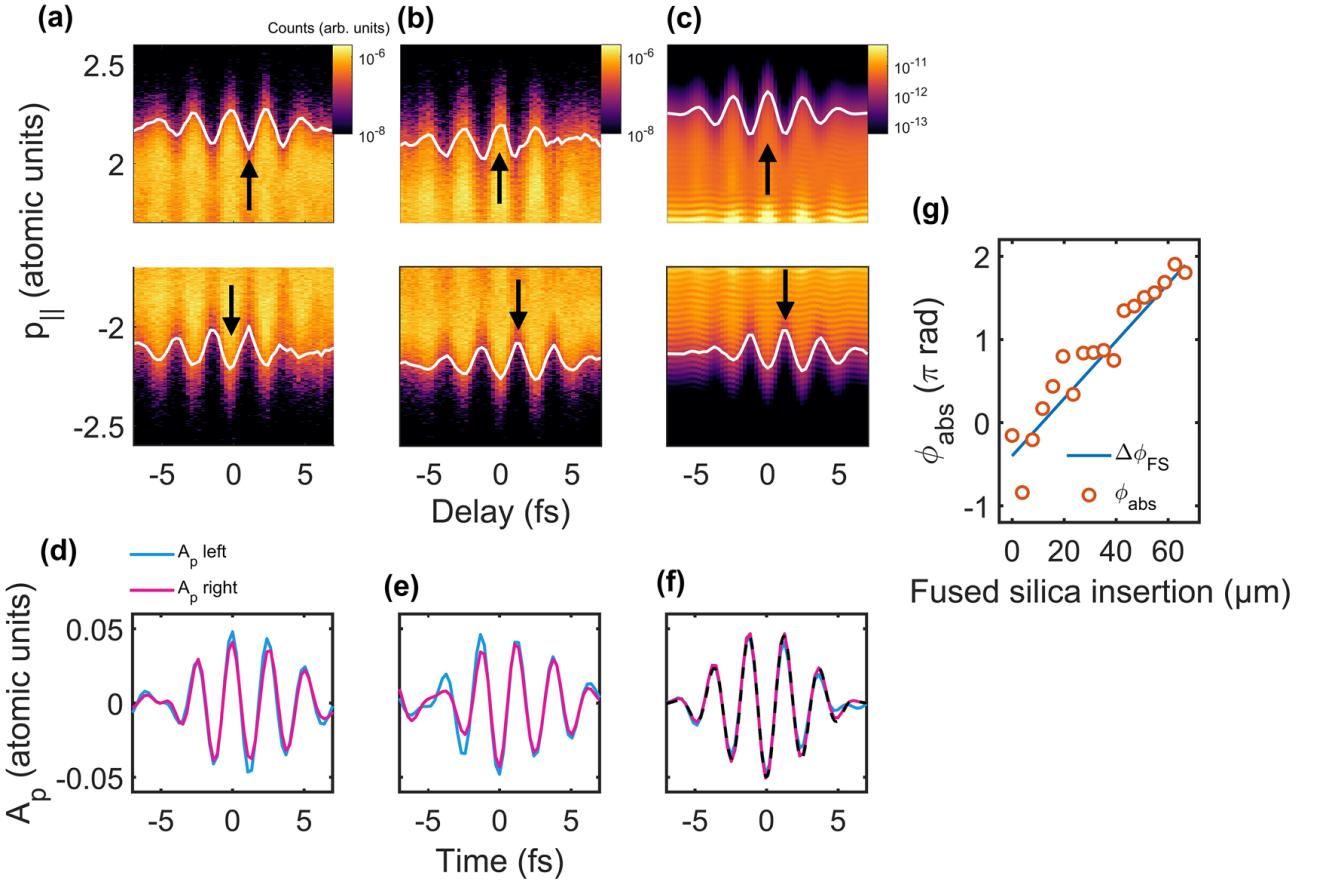
Complete characterization of the perturbing field using the ATI streaking method for different CEPs. (**a**–**c**) ATI streaking spectra for (**a**) experimental results with CEP equal to $-0.46\pi$, (**b**) $0.54\pi$, and (**c**) numerical results obtained by solving TDSE with the 5.2-fs pulse with CEP equal to $0.54\pi$, and a peak intensity of 1 × 10^–14^ W/cm^2^. ATI spectra in the focal volume were averaged for the numerical results. (**d**)–(**f**) Reconstructed temporal waveforms of vector potentials of perturbing fields using ATI spectra shown in each column. (**g**) The CEP of the reconstructed vector potential of the perturbing fields for different fused silica thickness. The CEP measured in the experiment (orange circles), and the theoretical estimation for a fused silica material (blue solid line) are shown.

The ATI spectra measured along the right (${p}_{||}>0$) and left ($p_{||} < 0$) directions are shown in the upper and lower panels, respectively, in Fig. 3a,b. They clearly show the modulation of the cutoff momentum as a function of time delay. The modulations in Fig. 3b are consistent with a numerical result shown in Fig. 3c obtained by solving the time-dependent Schrödinger equation in 1D for CEP equal to $0.54\pi$. The cutoff momentum shifts $\Delta p\left( \tau \right)$, which are denoted by white solid lines in Fig. 3a–c, were obtained by fitting the Airy function to the experimental data. A prominent feature is the half-cycle shift of the cutoff modulation observed along the two opposite directions and for two opposite CEPs as shown in Fig. 3a–c. The maximum amplitudes of the cutoff modulation are observed near the delay of 1.1 fs (black arrows) in the upper panel of Fig. 3a and the lower panels of Fig. 3b,c, while they are observed around -0.3 fs (black arrows) as shown in the lower panel of Fig. 3a and in the upper panels of Fig. 3b,c. The half-cycle shift of the cutoff modulation confirms that the electron liberated through tunneling at the single half-optical cycle contributes to the cutoff modulation along one direction, while the electron liberated at the next half-optical cycle contributes to the cutoff modulation along the opposite direction. These measurements confirm that the trajectories of the cutoff electrons liberated in a half-optical cycle of the main laser pulse can be successfully steered using the perturbing laser field.

The waveform of the perturbing laser field can be reconstructed from the cutoff momentum shift $\Delta p\left( \tau \right)$ using Eq. (1) (see the Methods section). The reconstructed waveforms obtained for both directions are consistent with each other as shown in Fig. 3d,f. The absolute phase of the waveforms is opposite for the two opposite CEPs as shown in Fig. 3d,e. We assigned the positive sign to the direction of the right arm of the stereo-ATI spectrometer and hence the positive (or negative) sign of the field corresponds to the field pointing toward the right (or left). To validate the accuracy of the reconstruction method, we reconstructed waveforms from the ATI spectra obtained numerically. The reconstructed waveforms for both directions (solid lines) are consistent with the original vector potential used in the numerical calculations as shown in Fig. 3f. In addition, we measured the waveform for different CEPs as shown in Fig. 3g. The CEP of the laser pulses was controlled by the wedge insertion thickness. The CEP shift expected from the theoretical values for a fused silica material is also shown in Fig. 3g. The slope of the two values, experimental and theoretical values, agrees well, showing the capability of the CEP measurement. Note that the absolute phase in Fig. 3g does not have the directional ambiguity in space, because the direction of the laser field can be fixed from the asymmetry measurement shown in Fig. 2.

**Figure 4:**
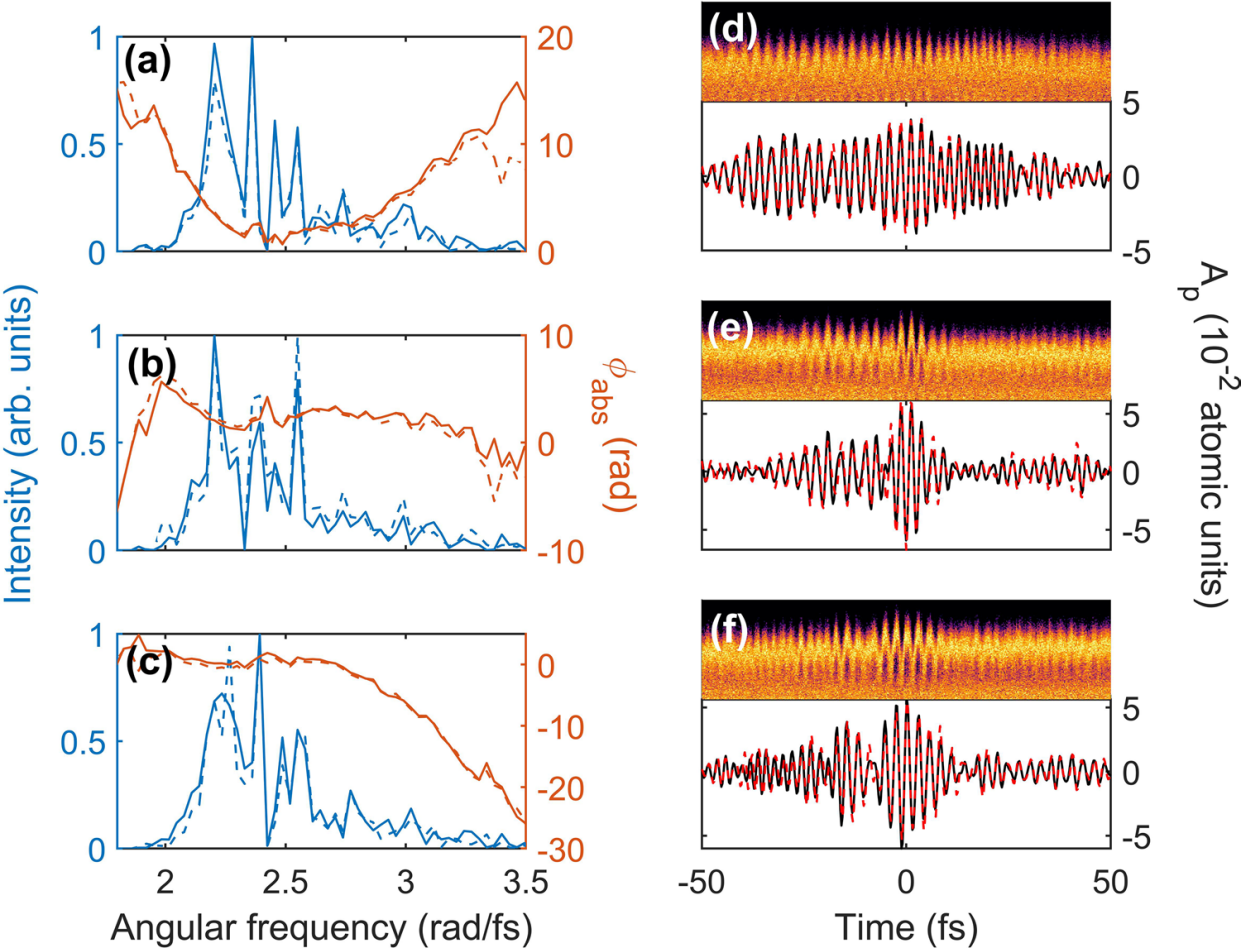
Spectra and temporal waveforms reconstructed by using the streaking method for different dispersions. (**a**)–(**c**) Spectra and absolute spectral phases for (**a**) positively chirped, (**b**) chirp free, and (**c**) negatively chirped pulses reconstructed by using ATI spectra recorded at the left (solid lines) and right (dashed lines). (**d**)–(**f**) Cutoff modulation of ATI spectra (upper panels for each subfigures) measured at the left arm of the stereo-ATI spectrometer and reconstructed temporal waveforms of (**d**) positively chirped, (**e**) chirp-free, and (**f**) negatively chirped pulses. The ATI spectra were measured as a function of delay, but it is shown here with the sign of the *x*-axis flipped and shifted in order to match the modulation with the temporal waveform. The temporal waveforms were reconstructed by using ATI spectra measured at the left (black solid line) and right (red dashed lines).

It is important to see if our approach is sensitive to the chirp of the laser pulse. For chirped laser pulse measurements, the main pulse should be fixed to the chirp free condition in which electrons liberated in a single half optical cycle contributes to the cutoff. The chirp of the perturbing laser field was controlled by a pair of glass wedges. Firstly, we performed a measurement with a chirp free perturbing laser pulse. Then, the ionization and rescattering times obtained for the chirp free perturbing laser pulse were used for the reconstruction of the chirped perturbing laser field (See the Methods section). The results of our measurements are summarized in Fig. 4. The reconstructed temporal waveforms are shown in Fig. 4d–f with corresponding ATI spectra shown in the upper panels. The spectral phases of the reconstructed pulses nicely exhibit the chirp structure of the positively chirped (Fig. 4a,d), chirp-free (Fig. 4b,e), and negatively chirped pulses (Fig. 4c,f) reconstructed for both the left (solid lines) and right (dashed lines) directions. The waveform measured along the two directions matches to each other even for their CEPs. We changed the fused silica insertion by $\pm$ 0.6 mm, and the reconstructed group delay dispersions (GDD) for positively chirped, chirp-free, and negatively chirped pulses were + 23.8 fs^2^, − 1.77 fs^2^, and − 28.1 fs^2^, respectively. The magnitude of the GDD difference between the positively chirped pulse and the chirp-free pulse is 25.6 fs^2^, and that between the chirp-free and the negatively chirped pulse is 26.3 fs^2^. The differences in GDD agree well with the expected GDD change 25.7 fs^2^ which can be calculated by group-velocity dispersion of fused silica. These results confirm the accuracy of our new streaking technique.

## Discussion

In summary, we demonstrated a new streaking method in which an electron liberated in an intense laser field is steered by adding a weak perturbing laser field. The addition of the perturbing laser field alters the cutoff energy of an ATI spectrum. The vector potential of the perturbing laser pulse has been successfully retrieved from the momentum shift of the ATI spectrum. The temporal waveform of the laser fields has been completely characterized, including its CEP, without directional ambiguity. The temporal waveforms of chirped pulses were also completely characterized, confirming the validity of our streaking method.

Our streaking technique can provide the laser field at the interaction region of above-threshold ionization, thus allowing in situ temporal characterization of a laser pulse. Since our approach can be combined with laser-induced electron diffraction experiments^18,21,22,28–31^ and it provides critical information on ionization and rescattering timings, it will become a versatile tool for analyzing the ultrafast electron dynamics of atoms and molecules in a strong laser field. The exact timing of the rescattering event and the accurate temporal profile of the laser pulse would provide important information in analyzing multi-electron effects in non-sequential double ionization^16,17^ and in time-resolved strong-field holography^20,32–34^.

## Methods

### Experimental methods

A CEP-stabilized, 1-kHz, 30-fs, 800-nm Ti:sapphire laser was used for our experiments. The laser pulse was compressed to 5 fs using a stretched-hollow-core fiber and a set of chirped mirrors. We placed a pair of wedges made of fused silica in the beam path and controlled the wedge insertion to control the CEP of the few-cycle laser pulse. An f-2f. interferometer setup was placed before the wedges and a feedback loop was made to stabilize long-term slow drift. The long-term standard deviation of the CEP of the compressed pulse was around 300 mrad. The beams were split into two and recombined by using a mirror with a round hole at the center at an angle of 45° with a diameter of 2.5 mm. The two beams are then focused into the stereo-ATI spectrometer by a spherical concave mirror with a focal length of 50 cm. In this way, the two pulses had the same temporal waveform at the focus. We measured ATI spectra for both left and right directions with a small opening angle (2°) along the polarization of the laser field. The target cylinder with two slits in the spectrometer was filled with Xe gas with a pressure of $2.0 \times 10^{ - 4}$ torr.

### Derivation of cutoff momentum shift ${\mathbf{\Delta p}}$ (Eq. 1)

The semi-classical trajectory model is used to derive Eq. (1). We consider an electron that tunnels out from an atom in an intense laser field. The electron is assumed to be driven by the laser electric field in dipole approximation $E\left( t \right) = {\text{d}}A\left( t \right)/{\text{d}}t$ after tunnelling ionization. Here, $A\left( t \right)$ is the vector potential of the laser field. The Coulomb field of the parent ion is neglected. The trajectories of the backward rescattered electron satisfy the rescattering condition, i.e. $\mathop \smallint \limits_{{t_{i} }}^{{t_{r} }} A\left( t \right)dt = \left( {t_{r} - t_{i} } \right)A\left( {t_{i} } \right)$, where $t_{r}$ is the backward rescattering time and ${\text{t}}_{{\text{i}}}$ is the corresponding ionization time.

The final momentum $p_{r}$ of the backward rescattered electron is $- 2A\left( {t_{r} } \right) + A\left( {t_{i} } \right)$. This final momentum is altered when a weak perturbing laser field is added to the main laser field. Because the perturbing laser field is weak, the final momentum of the electron can be expressed as

2 $$p_{r} \left( \tau \right) = - 2A_{m} \left( {t_{r} + \Delta t_{r} } \right) + A_{m} \left( {t_{i} + \Delta t_{i} } \right) - 2A_{p} \left( {t_{r} + \Delta t_{r} - \tau } \right) + A_{p} \left( {t_{i} + \Delta t_{i} - \tau } \right),$$

where $A_{m}$ and $A_{p}$ are the vector potentials of the main laser field and the perturbing laser field, respectively, and ${\tau }$ is the time delay between the two laser pulses. As the perturbing field is added to the main field, the rescattering and ionization times change slightly by $\Delta t_{r} \left( \tau \right)$ and $\Delta t_{i} \left( \tau \right)$, respectively. Each term on the right-hand side of Eq. (2) can be expanded into a Taylor series at $t_{r}$ (or $t_{i}$) because $\Delta t_{r}$ (or $\Delta t_{i}$) is small for a weak perturbing field. For example, we can approximate $A_{m} \left( {t_{r} + \Delta t_{r} } \right)$ as $A_{m} \left( {t_{r} } \right) - E_{m} \left( {t_{r} } \right)\Delta t_{r}$ and keep linear terms only. Thus, Eq. (2) can be rewritten as

3 $$p_{r} \left( \tau \right) = - 2A_{m} \left( {t_{r} } \right) + A_{m} \left( {t_{i} } \right) - 2A_{p} \left( {t_{r} - \tau } \right) + \frac{1}{{t_{r} - t_{i} }}\mathop \smallint \limits_{{t_{i} }}^{{t_{r} }} A_{p} \left( {t_{i} - \tau } \right){\text{d}}t$$

We did not drop electric field terms, for example, $E_{m} \left( {t_{r} } \right)\Delta t_{r}$, but they are cancelled by using a condition for $p_{r}$ to be extremum. Note that $t_{r}$ and $t_{i}$ are the rescattering and ionization times obtained in the absence of the perturbing field, respectively. Therefore, the shift $\Delta p$ in the cutoff momentum owing to the perturbing laser field can be written as Eq. (1).

### Reconstruction algorithm

We reconstructed the waveform of the perturbing field and the backward rescattering time using Eq. (1) in the main article. The waveforms of the main field and the perturbing field are assumed to be identical, but their amplitudes are different. Equation (1) in the main article can be viewed as a convolution between the spectral response function $S_{\omega }$ of the backward rescattering process and the vector potential of the perturbing field. The $S_{\omega }$ is a Fourier transform of the temporal response function $S_{t}$,

4 $$S_{t} \left( t \right) = - 2\delta \left( {t - t_{r} } \right) + \frac{{\Theta \left( {t - t_{i} } \right) - \Theta \left( {t - t_{r} } \right)}}{{t_{r} - t_{i} }},$$

where $\Theta$ is the Heaviside step function, and $\delta$ is the Dirac delta function. If we find $t_{i}$ and $t_{r}$, then we can find ${\text{S}}_{{\omega }}$ that can be used for the deconvolution to reconstruct the perturbing field.

Because $\Delta p\left( { - \tau } \right)/2$ is a good approximation of the vector potential, we use $\Delta p\left( { - \tau } \right)/2$ as an initial estimate for $A_{p} \left( t \right)$ and find $t_{i}$ and $t_{r}$ of the backward rescattered trajectory based on the semi-classical trajectory model. Then, a trial spectral response function $S_{\omega }$ using Eq. (4) is found and can be used for the deconvolution of $\Delta p\left( \tau \right)$. The next estimate of the vector potential can be found from the deconvolution result. We repeat the iterating process until the deconvolution results for $A_{p}$, $t_{i}$, and $t_{r}$ converge. Because $\Delta p\left( { - \tau } \right)/2$ is a good initial guess for the vector potential of the perturbing field the results converge rapidly within several iterations.

The minimum intensity of the perturbing laser field ($2 \times 10^{8}$ W/cm^2^) that can be measured can be estimated using the energy resolution of the TOF spectrometer (73 meV at 60 eV). The maximum intensity of the perturbing laser field that can be reliably measured were tested by solving the TDSE calculations for different intensities. These calculations showed that the intensity of the perturbing laser field should be weaker than 20% of the peak intensity of the main pulse.
